# A Patient With Unilateral Vocal Cord Paralysis Presenting to the Emergency Department With Voice Changes and Dyspnea

**DOI:** 10.7759/cureus.67845

**Published:** 2024-08-26

**Authors:** Richard Baluyot, Russell Mordecai, James Espinosa, Alan Lucerna

**Affiliations:** 1 Emergency Medicine, Jefferson Health, Stratford, USA

**Keywords:** management of vocal cord paralysis, diagnosis of vocal cord paralysis in the emergency department, causes of vocal cord paralysis, unilateral vocal cord paralysis, vocal cord paralysis

## Abstract

Unilateral vocal cord paralysis can cause a change in phonation and dyspnea and can be a cause of distress for a patient. The causes are varied and include post-surgical and post-intubation causes, malignancy, and other etiologies. Here, we present the case of a 72-year-old female who presented to the ED with a new onset of a "raspy voice" and dyspnea and had undergone an L4-L5 laminectomy with associated endotracheal intubation two weeks prior to ED presentation. Because of the complaint of a change in her voice, a nasopharyngolaryngoscopy (NPL) was performed, which demonstrated left-sided unilateral vocal cord paralysis. The patient was admitted to the hospital and was evaluated by neurology, pulmonology, and otolaryngology services. The discharge diagnosis was unilateral vocal cord paralysis, most likely caused by the patient's recent intubation. This case demonstrates the value of an NPL in the ED as part of the evaluation of a patient with dyspnea and a change in phonation.

## Introduction

There appears to be no clear data on the overall incidence of unilateral vocal cord paralysis. Concerning etiology, from 1985 to 1995, malignancy was the primary cause. However, this shifted to primarily iatrogenic surgically related injuries from 1996 to 2005 [1]. Post-intubation-related unilateral vocal cord paralysis can be a consequence of surgical procedures of all types; however, hyoid and thoracic surgery have been particularly linked to the risk of vocal cord paralysis. Idiopathic vocal cord paralysis has been described. Anterior approaches to the cervical spine and carotid endarterectomies, as well as traumatic injuries to the neck, have also been associated with unilateral and bilateral vocal cord paralysis. Malignancy continues to be a cause of unilateral vocal cord paralysis, including primary and metastatic lung carcinoma as well as thyroid tumors [1,2]. Neurologic causes of unilateral vocal cord paralysis can include stroke, multiple sclerosis, and myasthenia gravis. Sarcoidosis, varicella-zoster, and Lyme disease can also be causative [3]. Vocal cord paralysis can be caused by nerve damage or joint disorders [4]. Nerve damage involves the recurrent laryngeal nerve, which provides sensation to the glottis and sub-glottis, as well as motor innervation to the intrinsic muscles of the larynx responsible for abduction and adduction of the vocal cords. Therefore, any lesion, inflammation, or trauma to any aspect of the recurrent laryngeal nerve can cause unilateral vocal cord paralysis. Joint disorder-related vocal cord paralysis is related to dislocation or damage of the cricoarytenoid joint and/or cricothyroid joints. This can also be related to endotracheal intubation [5]. These are all causes of unilateral vocal cord paralysis that can be considered by an emergency physician.

This case report was presented in poster form at Rowan University Research Day, Stratford, NJ, on May 4, 2023.

## Case presentation

Here, we present the case of a 72-year-old female who presented to the ED complaining of dyspnea on exertion and a "raspy voice" with onset just prior to arrival. She had a history of chronic obstructive pulmonary disease as well as atrial fibrillation. The patient had undergone an L4-L5 laminectomy procedure approximately two weeks prior to the ED presentation. The procedure required endotracheal intubation. The patient denied chest pain, cough, rhinorrhea, fever, chills, or wheezing.

**Figure 1:**
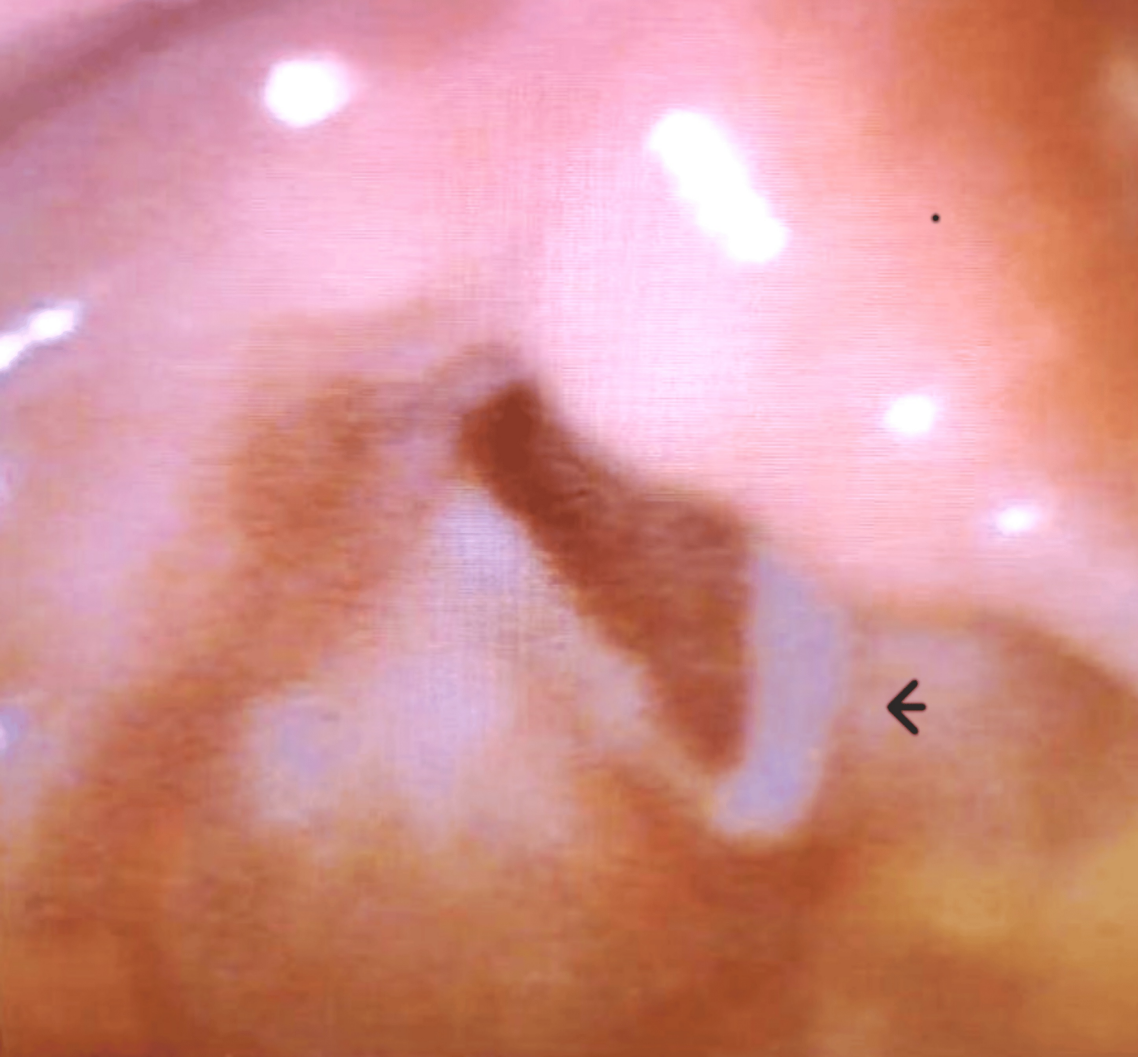
Image from NPL showing left vocal cord paralysis (arrow) NPL: nasopharyngolaryngoscopy

Vital signs were as follows: blood pressure 151/69 mm Hg, heart rate 130 beats per minute, respirations 25 breaths per minute, temperature 98.4°F, and pulse oximetry 98% on room air. The patient was awake and alert. The physical exam showed clear lung fields and was otherwise unremarkable. Because of the complaint of a change in her voice, a nasopharyngolaryngoscopy (NPL) was performed, which demonstrated left-sided unilateral vocal cord paralysis (Figure 1). The patient’s lab workup included a complete blood count, a basic metabolic panel, and troponin (Table 1). The results were within normal limits. A computerized tomographic angiogram of the chest showed findings consistent with chronic obstructive pulmonary disease but without any evidence of pulmonary embolism. An electrocardiogram showed normal sinus rhythm at 98 beats per minute and was within normal limits with no evidence of ischemia.

**Table 1:** Laboratory results BUN: blood urea nitrogen

	Result	Normal range	Units
White blood cell count	5.4	4.0-11.0	K/uL
Hemoglobin	11.0	10.6-15.6	g/dL
Platelet count	160.0	150-400	K/uL
Sodium	137.0	135-154	mEq/L
Potassium	3.6	3.5-5	mEq/L
BUN	18.0	5 to 20	mg/dL
Creatinine	1.1	0.6-1.2	mg/dL
Glucose	90.0	70-100	mg/dL
Calcium	10.0	8.5-10.5	mg/dL
Chloride	100.0	95-105	mEq/L
Bicarbonate	27.0	23-29	mEq/L
Troponin T (high sensitivity)	<2.3	<14	ng/L

The patient’s respirations improved to 18 breaths per minute without any intervention. However, the patient was still complaining of shortness of breath and a raspy voice change. The patient was admitted to the hospital and was evaluated by neurology, pulmonology, and otolaryngology services. The discharge diagnosis was unilateral vocal cord paralysis, most likely caused by the patient's recent intubation. The patient plan was outpatient follow-up with the otolaryngology service in one month for repeat NPL.

## Discussion

In this case, the left vocal cord was affected. The left laryngeal nerve is longer in its course than the right laryngeal nerve. This has been thought to be the reason that paralysis of the left vocal cord is up to 2.5 times more common than the right [5]. Compression of the recurrent laryngeal nerve can be related to the intubation process as well as to mechanical compression of the endotracheal tube [6].

In our case, the patient had a history of endotracheal intubation. The relative risk of intubation-related vocal cord paralysis increases in patients greater than 50 years of age, as well as in patients with diabetes and hypertension. The risk increases twofold in association with procedures greater than three hours of duration and 13-fold in procedures greater than six hours [4]. Surgery with intubation lasting greater than 12 hours was shown to have a 7% incidence of unilateral vocal cord paralysis in one single-center prospective study [7].

Our patient presented with a change in voice as well as dyspnea. Most patients who suffer from unilateral vocal cord paralysis of any cause will have symptoms consisting of a change in voice. Dyspnea has been reported in addition to phonation complaints [7]. A new onset of cough can be associated with vocal cord paralysis [8]. The clinical evaluation should include the past medical and surgical history. In addition, the time of onset and length of symptoms should be noted [7].

Because of a concern for vocal cord paralysis, an NPL was performed, which showed unilateral vocal cord paralysis. This was performed within an hour of the patient's presentation, which demonstrates that awareness of the possibility of unilateral vocal cord paralysis can be helpful in the ED. Direct visualization of the vocal cords can be achieved in such cases via NPL, which can be performed at the bedside, allowing direct visualization of the vocal cords and surrounding structures [4]. The workup can include a chest X-ray or CT scan of the chest for pulmonary causes and possibly a CT scan of the neck for soft tissue or infectious causes. CT scanning of the brain may be needed when neurological causes are a possibility [3].

Treatment of unilateral vocal cord paralysis is determined by the etiology of the paralysis. If the cause is damage to the recurrent laryngeal nerve, surgical interventions can be considered. Surgical interventions are varied and can include injection thyroplasty, in which fat or methylcellulose is injected close to the affected vocal fold, moving it medially to create better contact with the adjacent cord. Implants have been placed into the thyroid cartilage of the affected vocal cord in order to move it more medially. Laryngeal re-innervation surgery involves the movement of functioning nerves to the vicinity of the recurrent laryngeal nerve to reestablish tone and movement to the larynx [9].

Watchful waiting with otorhinolaryngology follow-up can be utilized with post-intubation-related vocal cord paralysis. This waiting period can be up to nine to 12 months [10]. Short-term oral steroids can be considered if there is swelling visible. Voice rehabilitation with speech and language therapists should always be included in treatment in order to improve glottic closure [6,7].

Laryngoelectromyography performed two months or more after symptom onset may have prognostic value [11]. Patients without complete resolution by nine months tend to have lifelong voice difficulties such as dysphonia [12]. In one study, the median time to full vocal cord recovery was approximately 74 days, with a maximum recovery of 482 days [7].

## Conclusions

Unilateral vocal cord paralysis can present with dyspnea and a change in voice, which can be distressing to a patient. Knowledge of the workup of such a patient, including the value of early NPL, can help in making the diagnosis and can initiate the process of identifying the underlying cause.
